# A Lightweight Sentiment Analysis Framework for a Micro-Intelligent Terminal

**DOI:** 10.3390/s23020741

**Published:** 2023-01-09

**Authors:** Lin Wei, Zhenyuan Wang, Jing Xu, Yucheng Shi, Qingxian Wang, Lei Shi, Yongcai Tao, Yufei Gao

**Affiliations:** 1 School of Cyber Science and Engineering, Zhengzhou University, Zhengzhou 450001, China; 2 Songshan Laboratory, Zhengzhou 450018, China; 3 School of Computer and Artificial Intelligence, Zhengzhou University, Zhengzhou 450001, China; 4 College of Intelligence and Computing, Tianjin University, Tianjin 300072, China

**Keywords:** sentiment analysis, global attention, multi-grained representation, lightweight

## Abstract

Sentiment analysis aims to mine polarity features in the text, which can empower intelligent terminals to recognize opinions and further enhance interaction capabilities with customers. Considerable progress has been made using recurrent neural networks or pre-trained models to learn semantic representations. However, recently published models with complex structures require increasing computational resources to reach state-of-the-art (SOTA) performance. It is still a significant challenge to deploy these models to run on micro-intelligent terminals with limited computing power and memory. This paper proposes a lightweight and efficient framework based on hybrid multi-grained embedding on sentiment analysis (MC-GGRU). The gated recurrent unit model is designed to incorporate a global attention structure that allows contextual representations to be learned from unstructured text using word tokens. In addition, a multi-grained feature layer can further enrich sentence representation features with implicit semantics from characters. Through hybrid multi-grained representation, MC-GGRU achieves high inference performance with a shallow structure. The experimental results of five public datasets show that our method achieves SOTA for sentiment classification with a trade-off between accuracy and speed.

## 1. Introduction

Opinion mining can be considered a particular sentiment classification task in natural language processing (NLP), which essentially learns semantic representations from unstructured texts and infers polarity. In recent years, the rapid growth of intelligent terminals has contributed to a boom in retail e-commerce networks, social services, and countless other applications [1], as well as further promoting the demand for opinion mining in intelligent interactions, such as smart customer service, intelligent robotics experts, etc. At the same time, online user-generated content, rich in personal sentiment and opinion, is being created and multiplying in social networks [2,3]. Detecting polarity based on these user-generated texts is crucial in various applications, including market fluctuations and decisions, product sales and feedback, political agendas, and polls [4]. In addition, sentiment analysis can contribute to the development of social psychology, social customer relationship management, political science, and other related disciplines on a large scale [5]. This commercial and innovative demand motivates researchers to explore sentiment feature computing.

Generally, sentiment analysis [6] can be regarded as a subfield of the text-classification problem. Almost all existing text-classification techniques are applicable to recognize sentiment. These methods can be subscribed to two categories: traditional machine learning [7] and deep learning [8,9]. Early research on sentiment analysis mainly focused on lexicon or rule-based machine learning methods. However, these methods employ only unigrams and bigrams of text features for matching and computation, without considering whole-sentence information. Therefore, they cannot achieve excellent performance owing to missing contextual information. In addition, these approaches rely on expensive manual operations, such as feature engineering, keyword annotation, and combating the effects of artificial subjectivity [10].

In recent years, deep learning methods have overcome their dependency on manual feature extraction and have yielded promising results in many tasks, including sentiment analysis, user intent mining, and content moderation [11,12,13,14]. Currently, prevalent approaches targeting sentiment analysis fall into two types. One is RNNs or CNNs with attention mechanisms to establish the connection between contextual contexts and critical polar features, which depend on several words in the whole sentence [15]. Other methods are pre-trained by large-scale corpora, such as Google’s BERT, which have been shown to be effective in improving performance on NLP tasks. Recently, BERT-based models [16] have been used to solve sentiment analysis tasks and achieved advanced accuracy.

However, it is challenging to efficiently deploy and run these advanced models on resource-limited micro-intelligent terminals because most current research revolves around accuracy; RNN-based, CNN-based, or BERT-based models are more complexly stacked. The sentiment analysis task for limited resources is not well studied and faces two key challenges: most minor memory consumption and highest inference speed [17]. (1) Smaller memory and storage are important constraints for embedded systems in micro-intelligent terminals. Nevertheless, although the BERT-based model achieves state-of-the-art performance, it consumes a large amount of memory. One disadvantage of BERT-based models, such as the basic BERT-base, is the large number of parameters (110 million), which can make them inappropriate for resource-constrained devices. Similarly, a large number of parameters are required in RNN-based models to fuel their sequence modeling capabilities [18]. (2) High latency reasoning can significantly diminish the user experience. The RNN-based models rely on a multitude of parameters for sequence modeling, and each of their inputs depends on the previous output, which limits parallelism and increases computational complexity. In contrast, the most current sentiment models based on BERT or CNN are deeply stacked structures, although they have parallel computing advantages. This causes them to require more computing power for reasoning, but it is not possible on micro-intelligent terminals.

To alleviate the above problems, this paper develops a lightweight sentiment analysis framework based on hybrid multi-grained embeddings to reduce computational cost while minimizing the loss of accuracy. First, global attention is designed to encode sentence contexts based on gated recurrent units, hoping to filter out noisy information. Second, multi-grained character information is utilized as a reference and supplement to further enhance the extraction of sensitive polarity representations in the downstream process. This work expects to enhance the robustness of the model by providing the richest possible embedding information while ensuring the model is as efficient as possible. Therefore, we suggest that it is feasible to construct a model with hybrid multi-grained embeddings to enable the deployment and inference of sentiment analysis tasks on devices. The main contributions in this paper can be summarized as follows.

(1) A lightweight and efficient sentiment analysis framework is proposed, which aims to consume memory and computational resources as least as possible, while accomplishing fast and accurate inference.
(2) To obtain accurate contextual semantics from unstructured text, we construct a shallow network layer by designing a global attention policy to filter contextual noise information with lower computational consumption.
(3) Furthermore, learning multi-grained character features provides richer semantic information to enhance the extraction of sentiment polarity features and improve the model’s overall performance downstream.
(4) The results of five public datasets show that our proposed reduces model size and has higher inference speed, reaching a state-of-the-art between accuracy and speed trade-off.

The remainder of this article is organized as follows. Section 2 briefly reviews related work. Our method is described in detail in Section 3. Section 4 provides the experimental results and Section 5 concludes our work.

## 2. Related Work

Early approaches to sentiment computing could usually be divided into two types: lexicon-based and traditional machine learning [19]. Lexicon-based methods contain primary information annotations for constituent words, phrases, or synsets [20,21]. There is a system of rules for polar, objective, intensive, and other complex semantic compositions to determine the sentiment orientation of text documents [22]. Although lexicons provide a valuable resource for archiving the affective polarity of words or phrases, using them to infer textual polarity is still quite challenging. No lexicon can address some of the semantic nuances observed from semantic compositionality or illustrate the semantic information of the context [23,24]. Therefore, some researchers have employed traditional machine learning based on statistical approaches to this field. In the development of sentiment analysis, machine learning approaches have employed a myriad of algorithms, including naive Bayes classifiers [25], nearest neighbors [26], and support vector machines [27], combined with features that range from bag-of-words (including weighted variants) [28]. Most of these methods rely on feature engineering that requires excessive manual operation. Despite best efforts, feature engineering can never be constantly updated and supplemented, which limits its generalization capability. As a result, deep learning methods have become the mainstream of sentiment analysis.

Deep learning has demonstrated superior performance in natural language over recent years, with the ability to model associations between contextual words [29,30]. In particular, the advent of the pre-trained model BERT has further prompted the sentiment analysis task [31]. More advanced models are often accompanied by more complex stacks and designs that are difficult to deploy at terminals, hindering the commercialization of artificial intelligence. Consequently, there needs to be more research on lightweight and efficient sentiment analysis tasks. The following three subsections present lightweight research on sentiment analysis.

### 2.1. Word-Based Methods

In general, word-based methods are based on continuous word representations, also known as word embedding [32,33], where each word is represented as a continuous low-dimensional vector to directly utilize the network. Kim [34] studied the application of the multi-channel convolution layers in sentence sentiment classification with promising results, and delved into the concept of non-static embeddings. Meanwhile, Kalchbrenner et al. [35] proposed a wide convolution model using k-max to retain more information in word tokens. Nevertheless, CNNs are only able to extract local features of the sequence, but the context is ignored. To incorporate contextual information, RNNs introduce an in-memory unit to retain a slice of crucial features so that long-distance dependencies between tokens can be captured. The classical modified type of RNNs, long short-term memory [36], and gated recurrent units [37], are currently the most popular networks used for text sentiment analysis. In addition, a sequential model with an attention mechanism has been effectively proved in sentiment analysis [38].

### 2.2. Character-Based Methods

Characters available significantly increase the amount of vocabulary the model can process and are flexible enough to deal with spelling errors and rare words. A full character-level neural translation model suggests that it can efficiently and resiliently learn semantic information that addresses out-of-vocabulary elements in sentences [39]. Similarly, Cherry et al. [40] determined that character-level translation models can substantially improve the precision of translations, especially for more complicated languages. Furthermore, characters can provide additional information about the text structure lost in word tokens [41]. The OpenAI team also reported that discovering sentiment features can be automatically captured while training an unsupervised model on the Amazon review corpus with characters alone [42]. However, characters do not have much meaning in English, despite mainly being the building blocks that make up words. Therefore, most of the existing character-based research is focused on complicated languages, such as Chinese.

Each character carries a specific meaning in Chinese, either as a word or as a phrase, which is different from English. Accordingly, learning Chinese characters extensively explores the semantic space and provides character-level sentiment information for sentiment analysis tasks. Wang et al. [43] utilized a parallel structure network for mining textual data with entirely Chinese characters. The model captured the semantic meaning using convolution and long short-term memory networks. Experiments showed that the dual-channel parallel structure outperformed the single-channel model. Furthermore, an attention mechanism was added to help the model perform better. In contrast, a character-based recognition model [44] for Chinese sentiment, which focuses on providing the most relevant and vital contextual words through pre-trained character encoding, also achieved excellent performance.

### 2.3. Word- and Character-Based Methods

Presently, pre-trained language models have become a research focus in sentiment analysis tasks. In natural language processing for Chinese tasks, most models adopt a mixture of words and characters for pre-training, since single characters also have specific semantic features, such as BERTs and RoBERTas [45]. The main characteristic of the pre-trained method is to train a highly generalized model based on a large corpus resource. Downstream tasks can then be easily fine-tuned by the generalized model to achieve significant results. However, the tiny version of ALBERT is widely employed due to the enormous size of the state-of-the-art pre-trained model and extremely expensive cost [46,47].

The tiny version is achieved by quantization, parameter sharing, and other tricks to compress the model size. Nevertheless, tiny versions still require significant resource consumption for pre-training and fine-tuning, which needs to be improved for smart terminals with limited memory and computing power. Considering the available open resources (pre-trained embeddings) are currently abundant enough, we expect to build an easy-to-use sentiment analysis framework on this basis. As for the already described approaches using public embeddings, most of them exploit more complex structural blocks for enriched semantics. Characters can provide unexpected implicit information, as mentioned in the previous section, but the feasibility of combining words and characters as information input has yet to be explored. Therefore, we desire to obtain richer textual information by fusing word and character features, so that more critical information can be learned and accuracy loss can be reduced, while designing a more lightweight network structure.

## 3. Method

**Figure 1:**
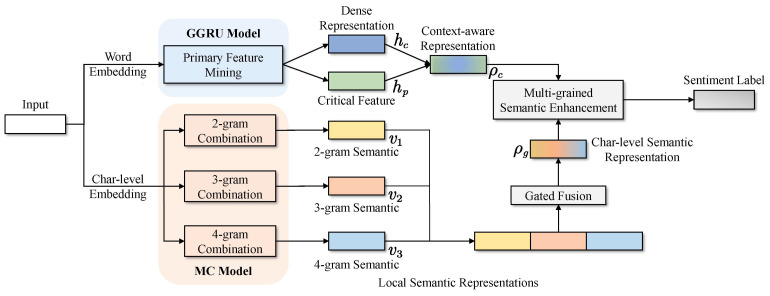
Overall architecture of our proposed hybrid multi-grained lightweight MC-GGRU.

The solution of the lightweight and efficient model designed in this paper is briefly illustrated in Figure 1. First, concerning word-level information, the global attention mechanism performs feature learning more efficiently without increasing too many parameters (Section 3.1). Second, multi-grained combination features learning character-level information obtain richer character information (Section 3.2). Finally, we fuse word- and character-level representations to further enhance the sensitivity of the downstream classifier to text polarity features (Section 3.3).

### 3.1. Words Modeling Layer

Consider a tokenized sentence $\left\{w_{1},\;w_{2},\;\ldots ,\;w_{n}\right\}$. The tokens are first converted into the corresponding word embedding $\left\{e_{1},\;e_{2},\;\ldots ,\;e_{n}\right\}$, where $e\in {\mathbb{R}}^{d}$, and d is the dimension of the embeddings. We model the token sequence using the GRU model, a variant of sequence models that changes the three gate units in the LSTM. The input and forget gates are merged into an update gate, and the output gate becomes a reset gate. Compared to LSTM, GRU has fewer parameters, faster convergence, and achieves superior performance on a specific-size dataset. When GRU is used to model input sequence, the hidden unit $h_{t}$ that each step-related feature representation can be calculated:

(1) $$h_{t}=GRU(e_{t},\;h_{t-1})$$

where $e_{t}$ is the current input text at the time t for GRU block and $h_{t-1}$ is the previous step hidden feature.

Although the basic GRU as an extractor balances the computation complexity of the whole model and improves the speed, the simple single-layer structure will lose some text information. In addition, the hidden state on each time step in the GRU contains the information only up to the current moment without synchronizing global information to calculate the sentiment relevance of each input, leading to the ignorance of some implicit polarity features defined by the context.

To balance the performance and complexity, inspired by [48], we design a global attention strategy to perceive local sentiment information through the global context to make token-level information modeling more accurate. Specifically, the relevance distribution of each input token to the sentiment context is calculated. Then, the original input information is sampled from the distribution as reinforcement features. The strategy is described in more detail below.

First, GRU learns the hidden state $h_{t}$ for each input text shown in Equation (1). It is assumed that the last hidden state $h_{n}$ is a contextual feature that can be used to obtain the contextual relevance distribution $p_{\alpha }$ for each token. Then, the original information that was activated is sampled from the distribution $z_{t}∼p_{\alpha }(1|e_{t},\;w_{t})$. The sampled information $z_{t}$ is incorporated into the sequence representation $h_{t}$, while all vectors are compressed into a dense vector d. The operation can be summarized as follows:

(2) $$p_{\alpha }(e_{t},\;w_{t})=\sigma (h_{n}^{⊤}\cdot W_{\alpha }(h_{t}))$$

(3) $$d=\sum \left(h_{t}⊕z_{t}\right)$$

where $h_{n}$ is the last hidden state, $W_{\alpha }$ is the parameter that the model needs to train, and d is the corresponding vector containing the primary sentiment features and the dense contextual representation. Finally, the text representation based on token modeling is as follows:

(4) $$\rho_{w}=ReLu(W_{w}\cdot d+b)$$

where ReLu is a linear activation function, $W_{w}\in {\mathbb{R}}^{d\times 2d}$ and $b\in {\mathbb{R}}^{d}$ are trainable parameters of the module.

In the word token modeling layer GGRU, we apply the basic sequence model to ensure that the model is small enough. Moreover, a global mechanism is designed to enhance feature extraction, leveraging only two linear layers without increasing many parameters and operations.

### 3.2. Multi-Grained Character Feature Layer

The multi-grained character feature layer MC aims to provide implicit features in the characters to enhance the robustness of the model [40,42]. Compared to English, individual characters are semantically richer in Chinese, and thus, can be well supported to run a full character-based model. Due to multiple combinations of Chinese characters, we extract multiple granularity semantic features based on characters. However, fully-character input sequences can be longer than tokens, resulting in dramatic parameter inflation utilizing the sequence model approach [18]. Considering the balance between model size and inference speed, the base multi-channel CNN is employed to model the character sequences. Under different sizes of shared convolutional kernels, multiple character combinatorial features are learned to reduce model parameters. The modeling process is described in detail below.

The input text is processed into a character encoding matrix $\left\{c_{1},\;c_{2},\;\ldots ,\;c_{m}\right\}$, $c_{i}\in {\mathbb{R}}^{d}$, where m represents the length of the sequence and d represents the vector dimension. First, different size filters are used to extract the local semantics $o_{i}^{\gamma }$:

(5) $$o_{i}^{\gamma }=f(W_{\gamma }\cdot c_{i:i+h-1}+b_{\gamma })$$

where $W_{\gamma }\in {\mathbb{R}}^{h\times d}$ is the trainable filter weight, $b_{\gamma }$ is the bias, $o_{i}^{\gamma }$ indicates values on the γ-th channel and i-th filter, and the filter size is h×d. Then, the optimal values are screened by the max technique. Finally, the filtered values are stitched together to represent one of the multi-granularity combinations based on characters. The features extracted by multiple filters are stitched to represent the multi-granularity information $\rho_{g}$ of the sequence, which can be represented by:

(6) $$m_{k}^{\gamma }=Max(o_{k,1}^{\gamma },\;o_{k,2}^{\gamma },\;\ldots ,\;o_{k,n-h+1}^{\gamma })$$

(7) $$v_{\gamma }=(m_{1}^{\gamma };\;m_{2}^{\gamma };\;\ldots ;\;m_{K}^{\gamma })$$

(8) $$\rho_{c}=W_{c}(v_{1};\;v_{2};\;\ldots ;\;v_{\gamma })$$

where Max is the max-pooling technique and K is the number of convolution filters.

In this step, parallel computation is optimized through convolution and filtering strategies to improve inference speed. Meanwhile, it generates structured feature mappings on characters, filtering and capturing semantic information on different granularity characters.

### 3.3. Hybrid Multi-Grained Learning

Instead of only utilizing words or characters, we provide text information using both token and character embedding. On the basis of the above two modules, feature learning is performed on both words and characters. Global attention is introduced to focus on keywords, and multi-granularity combination features are extracted on the character-level embedding. The pseudo-code for the model learning process is shown in Algorithm 1.

**Algorithm 1.** Hybrid multi-grained embedding-based lightweight model MC-GGRU**Require**: Training datasets with labels $X=\{x_{i},\;{\hat{y}}_{i}\}$, pre-trained embeddings, the maximum training iterations *T*.**Output**: Trained MC-GGRU sentiment classifier**Steps**:0: Initialize model parameters 1: **for**
*i* to *T* or until convergence **do**2:  **for**
*s∈minibatches S*
**do**3:   **for**
$e_{w}\in E_{w,s}$
**do**4:    Compute hidden states *h_t_ = GRU(e_w_)*5:    Token dense vector *d = global(h_t_)*6:    Sentence representation $\rho_{w}$7:   **end for**8:   **for**
$e_{c}\in E_{c,s}$
**do**9:    Multi-grained features *ρ_c_ = MC(e_c_)*10:   **end for**11:   **for**
_∈s
**do**
12:    *Enhancement Fusion(ρ_w_, ρ_c_)*13:   **end for**14:   Minimize the final loss function ℒ in Equation (10)15:  **end for**16: **end for**

The semantic word information $\rho_{w}$ with primary features captured by the GGRU module is fused with the local multi-grained semantics $\rho_{c}$ extracted by the MC to obtain the final representation. Finally, the representations are linearly transformed and fed into the classifier for classification.

(9) $$y=softmax(W(\rho_{c}⊕\rho_{g})+b)$$

where y is the prediction label, W are learnable parameters, and b is the linear bias term. $\hat{y}$ is set to be the actual data label. In this study, the backpropagation algorithm is used to optimize the model, and the loss function is:

(10) $$loss=-\sum_{i\in B}\sum_{j\in C}{\hat{y}}_{i}^{j}lny_{i}^{j}+\lambda ||\theta |{|}^{2}$$

where B is the size of the dataset, C is the data category, $\lambda ||\theta |{|}^{2}$ is a regularization term to prevent over-fitting.

To fully capture the semantic information in the text, both token and word embedding are used to provide information in the case of shallow structures. As a consequence, the model comprises two main layers, the word feature extraction layer and the multi-grain character feature layer. The former provides primary semantics, while the latter provides the latent semantics in characters. In the linear activation layer, the relu function is employed to optimally accelerate model training. The step-by-step flow is shown in Algorithm 1. The advantages of the approach proposed in this paper are of global attention for extracting primary information with context and entirely character-based feature learning that enhances the performance of the model. They will be discussed in the next section.

## 4. Results and Discussion

This section evaluates the proposed framework on five public datasets. They are user-generated content, including day life microblogs, product and hotel reviews, and social content regarding COVID-19. Each comment has a positive or negative label. The details of the dataset are shown in Table 1.

NLPCC 2013 and 2014: Both datasets were provided by Natural Language Processing and Chinese Computing (NLP&CC). The text in the corpus was collected from Sina Weibo with sentence-level tags divided into two categories based on positive and negative emotion labels. The content of the corpus was mainly related to users’ daily life, and the text was short with more popular network terms.

DF2020nCOV: A competition dataset for sentiment analysis of social comment related to the theme of COVID-19, jointly collected and organized by the China Computer Federation and Beijing Municipal Bureau of Economy and Information Technology at DataFountain. The corpus had a single theme, mainly around COVID-19 in early 2020, and the content was more serious than daily life.

Tan Songbo Hotel Reviews (TSBH): The corpus was collected and organized by professor Songbo Tan from reviews about hotels on the Ctrip application, and every review had a positive or negative label. The corpus has been widely used in studies for Chinese sentiment analysis [38].

TMALL: This corpus [43] had multiple domains and contained a variety of product-related reviews, such as books, home appliances, electronics, etc. These sentences were mainly collected from Tmall, accompanied by sentence-level tags. Similar to the previous one, the corpus content domain was relatively homogeneous.

### 4.1. Experimental Setup

For pre-processing, in the same sentence, all word tokens are segmented by Baidu Chinese segmentation API and all characters are obtained by jieba tool. Baidu stop word lexicon is leveraged to remove the stop word. In the experiments, all words and characters are initialized by wod2vec [49] pre-trained on an approximate 130 million Chinese Wikipedia corpus, and the dimension is 300. The words outside the vocabulary are randomly initialized in a uniform distribution. In addition, Adam Optimizer is employed to update the entire network in an end-to-end fashion.

During training, the critical parameters are searched as follows. The hidden state dimension is set to 256 for GGRU, and the convolution filter sizes are searched in ((1, 2, and 3), (2, 3, and 4), (3, 4, and 5), (1, 2, 3, and 4), (2, 3, 4, and 5), and (1, 2, 3, 4, and 5)) for character combinations in MC. The parameter learning rate and regular term coefficient are searched in (0.0001, 0.001. 0.01), and the mini-batch size is 100. Our experiments are executed on the Ubuntu operating system, using the PyTorch 1.8 framework and Python 3.6. The hardware includes an i7 CPU and a Tesla T4 GPU with 16G memory.

### 4.2. Baseline Methods

To comprehensively evaluate our proposed model on performance, we compared our approach with the following baseline models.

(1) MCNN [34] is a basic multi-channel convolutional neural network proposed for the text classification task application. The model performs a process similar to that of a single CNN, with the addition that the three optimal features obtained from each channel are finally concatenated to obtain more abundant and different granularity features. The sizes of the multiple filters are set to 3, 4, and 5, as configured by the authors.
(2) CGRU [50] is a single-channel hierarchical model for text information extraction, which performs local information mining by a single CNN and is followed by a GRU to learn the representation of the whole sequence.
(3) MC-AttCB [38] employs a complex structure for sentiment classification, which is the concatenation of a three-channel CNN and bidirectional GRU channel. Furthermore, a linear attention mechanism is integrated into the GRU and CNN, which is trained based on pre-trained word embeddings from a large-scale Wikipedia corpus. In contrast, this paper uses Wikipedia pre-trained embeddings while maintaining consistency in the other settings.
(4) T-CBGA [43] utilizes a parallel channel structure incorporating traditional CNN, GRU, and attention. Unlike our employment of characters as an aid, the model is entirely based on character encoding. The model is equipped with identical settings for both channels, and each channel performs 3-, 4-, and 5-gram feature extractions of the input character sequence by the MCNN. Then, the GRU with attention is used to train weights further for final classification. The model has achieved remarkable results in Chinese sentiment classification.
(5) CharBG [44] is a full character-level model for sentiment recognition in Chinese. A large-scale corpus collection is used to pretrain the character-level embeddings for the input sequence encoding, resulting in impressive performance. Due to data and hardware limitations, this paper applies an adaptive character embedding training strategy on the training corpus for the experimental setup.
(6) Glyph-CB [51] takes advantage of the characteristics of Chinese pictographs. The model learns multi-gram features, which are extracted by CNNs based on embeddings trained in a mixture of Chinese strokes and words. In addition, linear attention on the polarity features is also introduced. As the authors suggest, we adopt the same hyperparameter settings, except for the dimension of the input word vector.
(7) Albert-TZ [47] is an attention encoder network, a tiny version of pre-trained ALBERT with only four layers for Chinese. With the parameter-sharing strategy, Albert-TZ dramatically reduces the number of parameters and storage consumption compared to BERT without affecting performance.

### 4.3. Experiments and Results Analysis

#### 4.3.1. Overall Performance

In this subsection, we discuss different experimental results and analyze the advantages and disadvantages of our proposed model. The overall accuracy scores of our model compared to the baseline models on five corpora are presented in Table 2, and the results show:

The ALBERT-based model Albert-TZ shows the best performance on all corpora, demonstrating the effectiveness of the model pre-training with large-scale corpus. In addition, on the TMALL and DF2020nCOV corpora, Albert-TZ gains a more significant lead over other datasets. Specifically, compared to the model with the second highest performance, Albert-TZ improves higher 2.9%, 2.8%, 5.6%, 1.5%, and 2.6% accuracy scores on five datasets, respectively. It can be seen from Table 1 that the training corpus for NLPCC 2013, 2014, and TSBH is the minimum. Compared to other non-pre-trained models, Albert-TZ can significantly improve the accuracy of small datasets using pre-training parameters. Furthermore, there are more short texts in the NLPCC2013 corpus, and the pre-trained model has an impressive 3.7% higher score improvement.

The rest of the methods that do not use a pre-trained model can also obtain significant results. For example, in MCNN, CGRU, and MC-AttCB, favorable results have also been achieved using pre-trained word embeddings. In the TSBH dataset containing a large number of long sequences, we can observe that CGRU achieves better performance than MCNN. This result is because the longer the sequence, the richer the information GRU can capture. In contrast, the local limitation of CNN leads to some suitable solutions being discarded. With short textual datasets that contain less information, this is not the domain of the sequential model. In comparison, although MC-AttCB does not achieve better performance on TSBH, it achieves the highest accuracy score on DF2020nCOV. The overall structure of MC-AttCB is sophisticated after integrating the multi-channel attention mechanism. It could lay hold of more critical polarity features in the text, but also makes it weaker against a corpus with more sparse features (small and short), such as NLPCC2013.

For the character feature-based model, although the performance is the worst on CharBG, as shown in Table 2, it is only approximately 11.7% less compared to Abert-TZ on average. In addition, T-CBGA achieves second on individual datasets against the no-pre-trained model; for example, Tan Songbo Hotel Reviews. In comparison, the macro accuracy score of TCBA on all datasets is 5.8%, lower than SOTA. Its complex dual-channel cascading structure makes this possible, enabling it to retain more information as it learns. T-CBGA efficiently mines the advantages of CNN and GRU for a local feature and sequence-feature acquisition, respectively. The character feature-based model with such decent performance indicates the richness of textual information in character sequences, especially Chinese characters.

In Table 2, words- and glyph-based Glyph-CB achieved impressive accuracy scores on multiple corpora. Excluding SOTA Albert-TZ, Glyph-CB scored second in all three datasets, as well as in overall macro accuracy, just behind our proposed model. Moreover, compared to SOTA, Glyph-CB had only a 4.2% reduction in average macro performance. This result indicates abundant features in Chinese characters, and combining words and glyphs can generate more accurate polarity-aware information for classification. Compared to the base model MCNN, our model shows a maximum 2.2% improvement in performance. Moreover, our model improves by 1.4%, 0.9%, 1.1%, and 1.0% on the four datasets, compared with Glyph-CB. Although Glyph-CB uses word- and character-features as input, it relies more on glyph and stroke information in character features. In contrast, our model provides implicit semantic information directly through multi-granularity characters. It indicates that the multi-grained character layer in MC-GGRU can better provide richer polarity representations. Additionally, the global attention layer can also interactively learn contextual information and polarity-aware features, which further contributes to the performance of our model.

MC-GGRU is a relatively shallow network structure, performing poorly compared to the Albert-TZ model with more profound and complex blocks. However, on the dataset TSBH, MC-GGRU has only a 1.6% performance loss. This finding suggests that the structure of our model is effective and that decent performance can be achieved using only word embeddings. Although constructing increasingly complex network structures and parameter pre-training mechanisms can greatly facilitate achieving higher performance, it can also cause the model to contain more parameters and consume more computational resources, as described in Section 4.3.2.

#### 4.3.2. Model Statistics and Inference Speed

In order to gain more insight into the lightweight nature of our models, the size of each model is presented in Table 3. We used the thop toolkit to perform statistics on the TSBH (as can be inferred from Table 2), where the performance of all models differed the least. The parameters were the weight to be trained in a model, in other words, the variables needed to define the model. We took the same hyperparameters and ran them on the same hardware, Tesla T4.

It can be inferred from Table 3 that our model size is minimum besides the base model MCNN because our model is a shallower neural network than other models. The computational resource overhead is more critical on micro terminals than the model size storage consumption. MC-GGRU delivers a maximum 2.2% performance improvement with only a tenth increase in FLOPS, which is substantial. Compared with the third highest performer, Glyph-CB, our model consumes 0.46 MB less memory and 29% less computation, while a maximum of 1.4% improves the model performance. This indicates that our model’s resource consumption cost and performance are optimal in the non-pre-trained model. Although Albert-TZ is a tiny version of Albert, and the pre-training strategy enables Albert-TZ to perform best, its size is more than five times larger than MC-GGRU. Regarding computational cost, our model compresses the computation by a factor of 20 with a performance loss of only 3.3%. Consequently, our model is more applicable to micro terminals than the Albert-based model due to memory resource and computing power constraints. Therefore, better performance is delivered by deploying the proposed model on end devices, and the model explores the trade-off between performance and model size.

Inference speed is another essential metric to consider for neural networks. The inference speed can also be perceived as the computational efficiency of the network, and there are two primary evaluation criteria: throughput and responsiveness. The throughput represents the maximum rate of a network reasoning example, which can be further improved by optimizing the parallel processing strategy. The responsiveness can be expressed in the time used to reason regarding a model given a certain number of instances. The model can be made using parallel reasoning examples to have higher throughput and lower responsiveness. Therefore, in this paper, the only responsiveness is adopted as an evaluation metric for model inference speed to compare the computational cost of the models better.

As shown in Table 4, we count the responsiveness results of all models. The Albert-based model outperforms other models by a significant margin, having a more complex design and deeper stacked architecture. However, our model can achieve a 16x improvement in inference speed with a minimum reduction at 1.6% accuracy. Furthermore, compared to Glyph-CB, the second highest performing model in the baseline model, our model showed a maximum 1.4% improvement in accuracy and only a 0.04% decrease in responsiveness. Overall, our proposed model achieves an optimal trade-off between inference speed and performance.

### 4.4. Impact of Grained Size Selection

**Figure 2:**
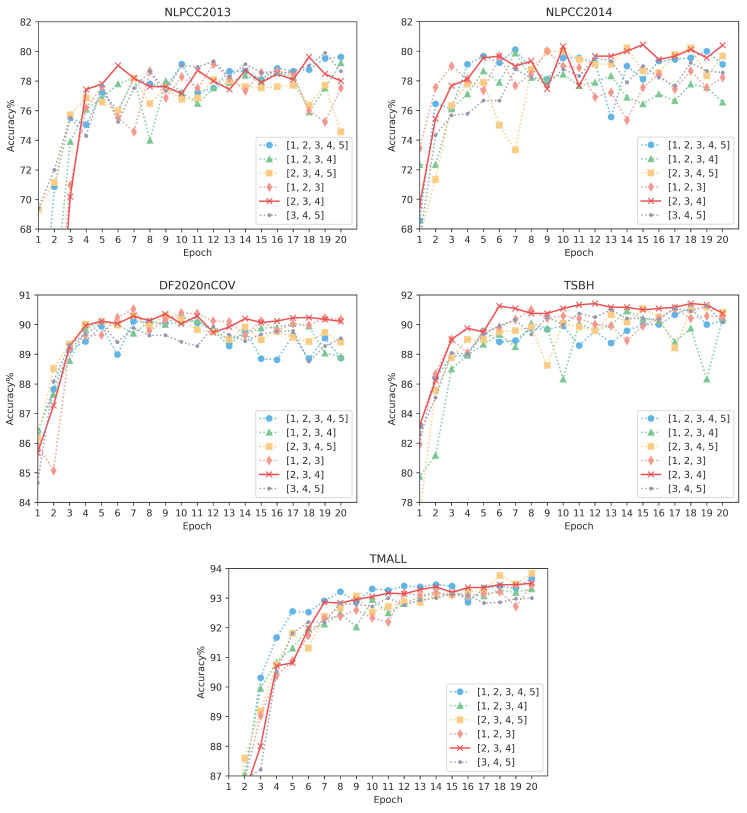
Sensitivity analysis for different granularity combinations on characters. These figures show the performance of the multi-grained feature layer using six combinations with twenty rounds trained on five datasets for each granularity choice.

Figure 2 reveals the performance of six granularity size selection mechanisms on our model. In Chinese, a single character is a word and can have various meanings in different combinations. According to Modern Chinese Latinized pinyin Chinese characters lexical statistics, most Chinese phrases consist of two to four words, so we use the multi-granularity combination (2, 3, and 4) as the primary choice for our model. To further verify the effectiveness of this combination, we compared six granularity combinations on five corpora, such as (1, 2, and 3), (2, 3, and 4), (3, 4, and 5), (1, 2, 3, and 4), (2, 3, 4, and 5), and (1, 2, 3, 4, and 5). The results of six granularity models running over twenty epochs on five datasets.

On the TSBH corpus with long text but a small total, it can be clearly seen that the granularity combinations (1, 2, and 3), (3, 4, and 5), and (2, 3, 4 and 5) all achieve significant accuracy scores, but (2, 3, and 4) converges more rapidly and steadily. Moreover, the three granularity options (1, 2, and 3), (1, 2, 3, and 4), and (1, 2, 3, 4, and 5) achieve excellent performance on NLPCC2013-2014 corpora. However, during our training, the combination with single-character combinations had difficulty converging several times. It can be speculated that is caused by the total corpus being too small and the text being short. The single-character meaning cannot be determined with the help of large amounts of data training and causes semantic confusion, eventually leading to the non-convergence of results. Nevertheless, (2, 3, and 4) also achieve excellent results with short texts. On the larger datasets (DF2020nCov, TMALL), it is evident that there is little difference in performance between several granularity combinations. In addition, although the result of (2, 3, and 4) is only marginally better, except for (1, 2, and 3), the model with granularity selection (2, 3, and 4) has fewer overall parameters and lower computational consumption. As a result, choosing (2, 3, and 4) is more favorable to accelerate the learning for our model without degrading the performance and keeping it stable.

### 4.5. Limitation

Although our proposed model has more advantages than other models, some areas still need to be focused on and addressed. There are several main defects.

First, our model usually produces a failed inference if there are both positive and negative opposite sentiment polarities in the sentence. Such sentences are frequently found in product reviews because there are many aspects of the product that can be evaluated, and which only sometimes receive several positive comments from users. As a result, our model sacrifices the ability of aspect-level sentiment analysis by focusing on lightweight. Second, the ability of inferencing vague expressions is far from ideal. For instance: the waiter should be kinder. Our model would incorrectly assume that the sentential expression is positive. Colloquial expressions cause this, and the model misses the background knowledge of colloquial common sense.

## 5. Conclusions

This paper proposes a lightweight and efficient sentiment analysis model MC-GGRU. It is built on a shallow structure to achieve high performance but with low complexity. The designed global layer can filter the noise to extract the crucial representations, while the multi-grained block further enriches downstream features for inferring sentiment with high computational efficiency. Extensive experiments are conducted on five datasets. Compared with the ALBERT-based SOTA method, MC-GGRU achieves a comparable performance, with less than a 5% loss in macro-average accuracy. Meanwhile, on TSBH, our model outperforms the pre-trained model in model size, computational consumption, and inference speed. Specifically, MC-GGRU reduces the size by more than five times and improves the inference speed by more than sixteen times, achieving an optimal trade-off between accuracy and speed. Moreover, in comparison with other lightweight non-pre-trained models, MC-GGRU reaches the best performance on four datasets. Therefore, it can be deployed on micro-intelligent terminals with limited memory and computing power for sentiment reasoning to provide a better user experience.

In the future, we will try to explore the following improvements. First, for some complex product reviews that contain multiple sentiments, it is difficult for MC-GGRU to identify the correct polarity. Future work could construct local attention strategies to capture the association between aspectual words and sentiment polarity while keeping the model as minimal as possible regarding resource consumption. Second, MC-GGRU needs to handle vague and ironic expressions well [52]. Therefore, we attempt to solve the problem of colloquial ambiguous or sarcastic expressions by integrating external knowledge in common sense into the model through transfer learning. Third, intelligent terminals are not limited to a single modal received in actual operation, such as voice and image. Whether multi-modal information [53] can be effectively fused will be the primary consideration in future lightweight model design. Finally, deploying effective and accurate models on embedded mobile devices is still challenging due to hardware resource constraints. The next step is further evaluating and improving our model by deploying them in actual micro terminals. We will further compress the model through quantization or pruning to improve the performance on the device.

## Figures and Tables

**Table 1:** The sentiment distribution of the adopted Chinese corpora.

No.	Corpus	Genre	Domain	Positive	Negative	Sum
1.	NLPCC2013	Microblog	Day life	2262	2675	4937
2.	NLPCC2014	Microblog	Day life	2700	2716	5414
3.	DF2020nCov	Microblog	COVID-19	16,899	17,121	34,020
4.	TSBH	Review	Hotel	3000	3000	6000
5.	TMALL	Review	Products	10,676	10,427	21,103

**Table 2:** The accuracy (%) on the test set for the Chinese corpus sentiment task. The models are divided into four categories: words-based, characters-based, and words- and characters-based. The bolded ones are the SOTA performance using the pre-trained model Albert, and the underlined ones are the best two performances for the non-pre-trained model.

Feature-Based	WORDS			CHARACTERS		WORDS + CHARACTERS		
Datasets	MCNN	CGRU	MC-AttCB	CharBG	T-CBGA	Glyph-CB	Albert-TZ	MC-GGRU
NLPCC2013	78.19	77.37	76.74	70.67	76.74	78.33	**82.31**	79.41
NLPCC2014	81.10	80.54	81.20	66.23	76.70	81.43	**84.23**	80.40
DF2020nCOV	89.24	89.34	89.62	85.04	88.96	89.42	**96.14**	90.46
TSBH	90.42	90.50	89.64	84.14	90.67	90.51	**93.19**	91.69
TMALL	91.76	92.69	92.15	88.26	91.72	92.82	**97.32**	93.79
Macro Avg.	86.14	86.10	85.87	78.87	84.96	86.50	**90.64**	87.15

**Table 3:** Model statistics for each model. Each model is evaluated on the TSBH corpus.

Models	Model Size		
	Parameters × 10^6^	Memory (MB)	FLOPS × 10^9^
MCNN	1.37	6.15	9.08
CGRU	3.71	16.59	22.09
MC-AttCB	4.82	18.13	30.33
CharBG	6.89	32.82	7.29
T-CBGA	2.79	13.82	10.46
Glyph-CB	1.99	9.60	13.44
Albert-TZ	11.89	47.58	210.79
MC-GGRU	1.81	9.14	10.44

**Table 4:** The relative processing speed compared to our proposed model after five repetitions on the inference process.

Models	Model Responsiveness	
	Mean (%)	Std
MCNN	0.91	0.0021
CGRU	0.77	0.0157
MC-AttCB	2.49	0.0310
CharBG	0.51	0.0040
T-CBGA	5.68	0.0233
Glyph-CB	0.96	0.0064
Albert-TZ	16.27	0.3055
MC-GGRU	1.00	—

## Data Availability

The data presented in this study are openly available in [http://tcci.ccf.org.cn/conference/2013/pages/page04_tdata; http://tcci.ccf.org.cn/conference/2014/pages/page04_tdata; https://www.datafountain.cn/competitions/423/datasets; http://www.searchforum.org.cn/tansongbo/corpus, accessed on 4 December 2022].
